# Beta-lactam dosing during continuous renal replacement therapy: a survey of practices in french intensive care units

**DOI:** 10.1186/s12882-022-02678-x

**Published:** 2022-01-29

**Authors:** Elodie Matusik, Justine Lemtiri, Guillaume Wabont, Fabien Lambiotte

**Affiliations:** 1 Department of Pharmacy, Valenciennes General Hospital, Valenciennes, France; 2 Department of Intensive Care Unit, Valenciennes General Hospital, Valenciennes, France

**Keywords:** Beta lactams, Pharmacokinetics, Renal replacement therapy, Critical illness, Surveys and questionnaires

## Abstract

**Background:**

Little information is available on current practice in beta-lactam dosing during continuous renal replacement therapy (CRRT). Optimized dosing is essential for improving outcomes, and there is no consensus on the appropriate dose regimens. The objective of the present study was to describe current practice for beta-lactam dosing during CRRT in intensive care units (ICUs).

**Methods:**

We conducted a nationwide survey by e-mailing an online questionnaire to physicians working in ICUs in France. The questionnaire included three sections: demographic characteristics, CRRT practices, and beta-lactam dosing regimens during CRRT.

**Results:**

157 intensivists completed the questionnaire. Continuous venovenous hemofiltration was the most frequently used CRRT technique, and citrate was the most regularly used anticoagulant. The median prescribed dose at baseline was 30 mL/kg/h. The majority of prescribers (57%) did not reduce beta-lactam dosing during CRRT. The tools were used to adapt dosing regimens during CRRT included guidelines, therapeutic drug monitoring (TDM), and data from the literature. When TDM was used, 100% T > 4 time the MIC was the most common mentioned pharmacokinetic/pharmacodynamic target (53%). Pharmacokinetic software tools were rarely used. Prolonged or continuous infusions were widely used during CRRT (88%). Institutional guidelines on beta-lactam dosing during CRRT were rare. 41% of physicians sometimes consulted another specialist before adapting the dose of antibiotic during CRRT.

**Conclusions:**

Our present results highlight the wide range of beta-lactam dosing practices adopted during CRRT. Personalized TDM and the implementation of Bayesian software appear to be essential for optimizing beta-lactam dosing regimens and improving patient outcomes.

**Supplementary Information:**

The online version contains supplementary material available at 10.1186/s12882-022-02678-x.

## Background

Beta-lactams are the most widely prescribed antibiotics in critically ill patients. Optimized dosing of beta-lactams is required to deal with pharmacokinetic changes and frequent underdosing – especially during the early phase of sepsis [1–3]. Renal failure may protect patients from insufficient antibiotic exposure by increasing the probability of beta-lactam target concentration attainment [2–5], and patients may benefit from high doses – particularly during the first 24 to 48 h of antibiotic treatment [3, 6]. Several experts have called for caution when using reduced dosing regimens in patients receiving continuous renal replacement therapy (CRRT) [7, 8]. Seyler et al. demonstrated the inadequacy of the recommended beta-lactam dosing regimens during CRRT when bacteria with a high minimal inhibitory concentration (MIC) are involved [9]. Underdosing has prompted several experts to suggest the use of non-adjusted dosing within the first 24 to 48 h [10–14], albeit with a potential risk of overdose [12–15]. Although beta-lactam dosing can be guided by clinical and pharmacokinetic data from the literature, the studies concern mainly intermittent hemodialysis and cannot be applied to CRRT, for which a variety of practices are used [15, 16]. Li and Vaara highlighted the lack of key information required to correctly interpret studies and devise dose adjustments [18, 19]. In 2019, these marked differences in CRRT practices and the subsequent influence on beta-lactam elimination prompted two French learned societies to recommend therapeutic drug monitoring (TDM) of beta-lactams in patients receiving CRRT [5]. In 2020, several international learned societies recommended TDM for routine use in critically ill patients [20]. However, TDM is not often available outside university hospitals. Given these uncertainties and our experience that neurotoxicity is more frequent in patients receiving CRRT, we decided to survey beta-lactam dosing practices during CRRT in France.

## Methods

### Survey development

Given the absence of data on the beta-lactam dosing prescribed during CRRT in France, we designed a survey to assess current practices. It was developed by a pharmacy resident with help from a critically ill clinical pharmacist and an intensivist, after a review of the literature. We performed an online, nationwide, cross-sectional survey between July and September 2019 by emailing a questionnaire to 1423 senior physicians working in intensive care units (ICUs) across France. The survey included 22 questions on the respondents’ characteristics, CRRT practices, and beta-lactam dosing regimens during CRRT. The English version of the questionnaire and the results are given in Table 1. In order to determine which membrane material was used, brand names were cited in the questionnaire. Three clinical vignettes describing a critically ill patient weighing 70 kg and being treated for infectious pneumonia with piperacillin-tazobactam, cefotaxime or meropenem were used to prompt respondents to describe their beta-lactam dosing practices during CRRT (at 25 ml/kg/h). The questionnaire was made available on Google Forms® (Google, Inc., Mountain View, CA, USA). Data were extracted into an Excel® spreadsheet (Microsoft Corp, Redmond, WA, USA). Participation was anonymous. According to French legislation, approval by an investigational review board was not required for this survey.

**Table 1:** The survey results

Questions	Responses are n/N (%) unless otherwise indicated
**Demographic characteristics**	
What is your medical qualification?	
Critical care medicine	77/157 (49)
Anesthesiology	61/157 (39)
Pulmonology	5/157 (3)
Nephrology	5/157 (3)
Emergency	4/157 (3)
Internal medicine	2/157 (1)
Cardiology	2/157 (1)
Infectious disease	1/157 (1)
For how many years have you worked in an ICU (years, median [IQR])	10 [4-18]
In which type of institution do you work?	
University hospital	78/157 (50)
Public-sector general hospital	71/157 (45)
Private-sector for-profit or non-profit hospital	8/157 (5)
**CRRT practices**	
Which RRT modality do you most commonly use?	
Continuous renal replacement therapy	110/157 (70)
Intermittent renal replacement therapy	47/157 (30)
Which CRRT techniques do you use?	
Continuous venovenous hemofiltration	114/157 (71)
Continuous venovenous hemodialysis	89/157 (57)
Continuous venovenous hemodiafiltration	87/157 (55)
Sustained Low-Efficiency Dialysis	13/157 (8)
Which is the most commonly used CRRT technique?	
Continuous venovenous hemofiltration	81/156 (52)
Continuous venovenous hemodialysis	54/156 (35)
Continuous venovenous hemodiafiltration	21/156 (13)
Which is the most commonly used anticoagulant?	
Citrate	100/157 (64)
Heparin	57/157 (36)
If you use continuous venovenous hemofiltration or hemodiafiltration, which hemofiltration mode do you prefer?	
Postdilution mode	22/142 (16)
Predilution mode	11/142 (8)
Pre/postdilution mode	106/142 (77)
Which is the most commonly used CRRT dose, and how do you adjust it?	
20 mL/kg/h	9/157 (6)
25 mL/kg/h	36/157 (23)
30 mL/kg/h	49/157 (31)
35 mL/kg/h	45/157 (29)
2000 mL/h (effluent flow not adjusted for body weight)	12/157 (8)
2500 mL/h (effluent flow not adjusted for body weight)	3/157 (2)
3000 mL/h (effluent flow not adjusted for body weight)	3/157 (2)
Dialysis dose adjusted for body weight upon CRRT initiation	29/75 (39)
Dialysis dose adjusted for body weight on admission	27/75 (36)
Dialysis dose adjusted for ideal total weight	19/75 (25)
Which type(s) of membrane do you use for CRRT?	
Polysulfone (Fresenius® kits: CVVHDF 600, CVVHDF 1000, CVVH 600, CVVH 1000, HV-CVVH 1000, Ci-Ca postCVVHDF 1000, Ci-Ca CVVHD 1000, Ci-Ca EMiC2 ; Theradial® kits: Aquamax HF12, HF 19)	54/138 (39)
Polyarylethersulfone (Baxter® kits: Prismaflex HF1000, HF1400)	9/138 (7)
Acrylonitrile (Baxter® kits: Prismaflex M100, M150)	29/138 (21)
Acrylonitrile coated with polyethylenimine (Baxter® kits: Prismaflex ST100, ST150)	55/138 (40)
**Beta-lactam dosing regimens during CRRT**	
Which beta-lactam dosing regimen do you prescribe for patients on CRRT?	
Unadjusted dosing regimens	88/157 (56)
Full dose for 24 h and then a reduced-dosing regimen	26/157 (17)
Full dose for 48 h and then a reduced-dosing regimen	14/157 (9)
A single loading dose before a reduced-dosing regimen	20/157 (13)
Reduced-dosing regimens all the time	3/157 (2)
Reduced or full doses, depending on the drug compound	6/157 (4)
Do you adjust the antibiotic dose based on the dialysis dose or effluent flow?	
Yes	23/154 (15)
No	131/154 (85)
Do you use prolonged/continuous infusions for beta-lactams in patients on CRRT?	
Yes	138/157 (88)
No	19/157 (12)
If yes, for which beta-lactam?	
Piperacillin/tazobactam	108/119 (91)
Cefotaxime	63/119 (53)
Ceftazidime	85/119 (71)
Cefepime	59/119 (50)
Meropenem	47/119 (40)
If yes, which tools do you use?	
* Guide Prescription et Rein* (French renal prescription handbook)	103/139 (74)
Therapeutic drug monitoring	86/139 (62)
Data from clinical studies in the literature	48/139 (35)
Dosing regimens of patients with renal failure applied to the estimated creatinine clearance rate of the patient on RRT	6/139 (4)
Pharmacokinetic software	4/139 (3)
Pharmacokinetic calculations by hand	3/139 (2)
Other tools	6/139 (4)
If beta-lactam therapeutic drug monitoring is used, which pharmacokinetic/pharmacodynamic target do you use?	
40/50/70% T > MIC	0/74 (0)
100% T > MIC	12/74 (16)
40/50/70% T > 4 MIC	4/74 (5)
100% T > 4 MIC	39/74 (53)
40/50/70% T > 5 MIC	0/74 (0)
100% T > 5 MIC	7/74 (10)
40/50/70% T > 8 MIC	0/74 (0)
100% T > 8 MIC	12/74 (16)
Do you sometimes call other specialists for advice on antibiotic dosing regimen adjustment for patients on CRRT?	
No	92/157 (59)
Infectious disease specialist	45/157 (29)
Microbiologist	16/157 (10)
Pharmacist/pharmacologist	13/157 (8)
Nephrologist	8/157 (5)
Toxicologist	8/157 (5)
For a 70 kg patient admitted with community-acquired infectious pneumonia and treated with your preferred CRRT technique at 25 ml/kg/hour, which maintenance dose do you prescribe for cefotaxime?	
2 g TID	84/156 (54)
2 g BID	17/156 (11)
1 g TID	44/156 (28)
1 g BID	9/156 (6)
1 g QID	2/156 (1)
For a 70 kg patient admitted with hospital-acquired infectious pneumonia and treated with your preferred CRRT technique at 25 ml/kg/hour, which maintenance dose do you prescribe for piperacillin/tazobactam?	
4/0.5 g QID	61/154 (40)
4/0.5 g TID	69/154 (45)
4/0.5 g BID	15/154 (10)
3/0.375 g QID	6/154 (4)
Other	2/154 (1)
For a 70 kg patient admitted with hospital-acquired infectious pneumonia and treated with your preferred CRRT technique at 25 ml/kg/hour, which maintenance dose do you prescribe for meropenem?	
2 g TID	48/156 (31)
2 g BID	9/156 (6)
1 g TID	69/156 (44)
1 g BID	28/156 (18)
Other	2/156 (1)
Does your institution have procedures for adjusting antibiotic doses in patients on CRRT?	
Yes	33/157 (21)
No	124/157 (79)
Do you feel that you observe more beta-lactam-induced neurotoxicity in patients treated with renal replacement than in other patients?	
Fully agree	5/157 (3)
Tend to agree	48/157 (31)
Tend to disagree	80/157 (51)
Strongly disagree	24/157 (15)

Abbreviations: *CRRT* continuous renal replacement therapy, *MIC* minimum inhibitory concentration, *BID* twice a day, *TID* three times a day, *QID* four times a day

### Statistical analysis

The results were presented as the frequency (percentage) for qualitative variables and the median [interquartile range (IQR)] for quantitative variables. For statistical comparisons of different groups, we applied Pearson’s chi-square test with Yates’ correction. All tests were two-sided, and the threshold for statistical significance was set to p<0.05. Statistical tests were performed using SAS® software (version 3.8, SAS Institute, Cary, NC, USA).

## Results

### The respondents’ characteristics

Of the 1423 physicians contacted, 157 (11%) replied. Physicians working in university hospitals accounted for 50% of the respondents, whereas 45% of the respondents worked in public-sector general hospitals and 5% worked in private for-profit or non-profit hospitals. They had a median of 10 years [4–18] of experience in the ICU. Most of the physicians had trained in critical care medicine (49%) and anesthesiology (39%).

## CRRT practices

Concerning renal replacement therapy (RRT), CRRT was preferred to intermittent hemodialysis (70%). The CRRT techniques used by intensivists were variously venovenous hemofiltration (73%), venovenous hemodiafiltration (57%), venovenous hemodialysis (55%), and Sustained Low-Efficiency Dialysis (8%). Half of the physicians (52%) reported prescribing continuous venovenous hemofiltration preferentially, followed by continuous venovenous hemodialysis (35%). 64% of the respondents used citrate as the anticoagulant. 77% of the physicians prescribing hemofiltration reported using a combined predilution and postdilution modality. The median prescribed dose at initiation was 30 mL/kg/h, and 75% of the prescribers considered the total body weight (with 39% for body weight on admission and 36% for body weight on the day of the CRRT prescription). 12% of the respondents reported prescribing a flow effluent irrespective of body weight. Polyacrylonitrile was the most commonly used membrane material (61%, 40% of which were polyethylenimine-coated), followed by polysulfone (39%).

## Beta-lactam dosing regimens during CRRT

**Fig. 1:**
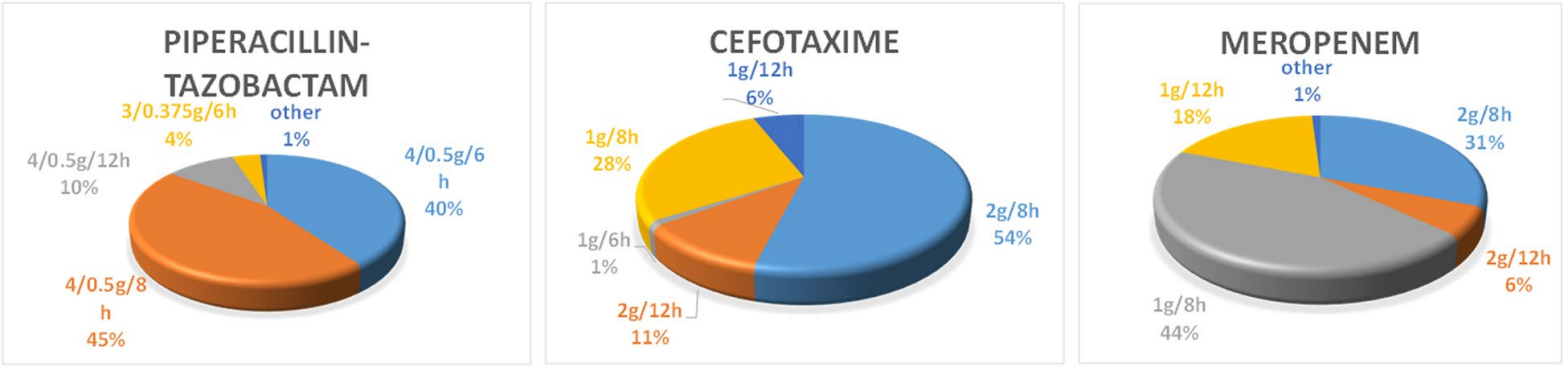
Summary of responses to the clinical vignettes in the study questionnaire

Concerning beta-lactam prescriptions during CRRT, the majority of the physicians (56%) did not adjust the doses. 17% and 9% of them prescribed full doses for 24 and 48 h, respectively, before reducing the dosing regimens. 13% of respondents reported prescribing a single loading dose before dose adjustment and 4% reported that their use of a reduced dose or a full dose depended on the antimicrobial agent in question. Only three physicians reported using reduced doses all the time. A dose adjustment could be either empirical or adapted according to the TDM results. In 85% of cases, the respondents did not adapt the beta-lactam dosing as a function of the CRRT dose or the effluent flow (85%). The physicians used mainly the French renal prescription handbook (*Guide Prescription et Rein*) (74%), TDM (62%), and data from the literature (35%) to adjust the beta-lactam dosing regimens. Only 4 physicians reported using pharmacokinetic software tools. When TDM was used, 100% T > 4 MIC was the most common pharmacokinetic/pharmacodynamic (PK/PD) target. 41% of the respondents sometimes consulted another specialist when deciding whether or not to adjust the beta-lactam dose during CRRT: this was variously an infectious disease specialist (29%), a microbiologist (10%), a pharmacist/pharmacologist (8%), a nephrologist or toxicologist (5%). The replies to the clinical vignettes highlighted a broad range of dose adaptation practices - particularly for meropenem (Fig. 1). Most participants used prolonged and continuous infusions (88%), especially for piperacillin-tazobactam (91%), ceftazidime (71%), cefotaxime (53%), cefepime (50%) and meropenem (40%). Only 21% of physicians reported having access to a procedure for determining beta-lactam dosing regimens during CRRT. Only 34% of the physicians had the feeling that neurotoxicity was more frequent during CRRT. The use of TDM was significantly associated with prolonged and continuous infusions (*p*=0.016) and a call to other specialists for advice on antibiotic dosing regimen adjustment during CRRT (*p*<0.0001) (Table 2).

**Table 2:** Factors associated with the use of therapeutic drug monitoring

	Use of therapeutic drug monitoring			
		**Yes** ***n* = (%)**	**No** ***n* = (%)**	***p*-value**
**Type of institution**				
University hospital		45	33	0,569
Other types of hospital		41	38	
**Years of experience ≤ 10**				
Yes		48	48	0,493
No		38	29	
**No adjustment of dosing regimens during CRRT**				
Yes		48	45	0,425
No		38	26	
**Do you adjust the dosage according to the dialysis dose or effluent flow?**				
Yes		13	10	1,000
No		72	59	
**Do you call other specialists for advice on antibiotic dosing regimen adjustments for patients on CRRT?**				
Yes		47	18	**<0,001**
No		18	53	
**Does your institution have procedures for adjusting antibiotic doses in patients on CRRT?**				
Yes		17	16	0,821
No		69	55	
**Do you use prolonged/continuous infusions for beta-lactams in patients on CRRT?**				
Yes		81	57	**0,016**
No		5	14	

The various groups were compared using Pearson’s chi-squared test with Yates’ correction. The threshold for statistical significance was set to *p*<0.05

## Discussion

To the best of our knowledge, the present study is the first to have assessed beta-lactam dosing practices in the context of CRRT. Furthermore, the survey described CRRT practices in France. Physicians practicing intensive care medicine (whatever their initial qualification) were included in the survey. The majority were intensivists or anesthesiologists, which reflects the fact that anesthesiologists are qualified for critical care medicine in France.

In order to review beta-lactam dosing in CRRT, we analyzed compliance with the French guidelines and Kidney Disease: Improving Global Outcomes (KDIGO) guidelines [21, 22]. Our present findings were in line with studies of CRRT practices performed over the last decade [23–25]. CRRT was preferred to intermittent hemodialysis, although there is no clear evidence of superiority concerning reduced mortality [21, 22]. This technique is considered to provide greater hemodynamic stability. As recommended, citrate was the main anticoagulant used [21, 22]. Multicenter randomized controlled trials and meta-analyses have shown that increasing the CRRT dose intensity above 20-25 ml/kg/h does not increase survival but does lead to more metabolic complications [26–29]. Therefore, in order to deliver a dose of 20–25 ml/kg/h and to minimize interruptions in the CRRT, the KDIGO guidelines recommend a value of 25–30 ml/kg/h [22]. Although citrate limits filter coagulation, a third of our respondents indicated (as also found in other studies) that they prescribe a higher CRRT dose - leading to greater clearance of beta-lactams [23, 25]. The physicians reported using different reference body weights to prescribe the CRRT dose, which contributed to disparities in RRT practices. Although most studies are based on total weight at the time of randomization, the guidelines do not specify the weight to be used to determine the dialysis dose - resulting in a variety of practices. Our survey highlighted the diversity and lack of harmonization of CRRT techniques.

Physicians may not be sufficiently aware of the need to maintain beta-lactam full doses during the initial phase of treatment. Administration must be optimized in order to maintain effective antibiotic concentrations for a sufficiently long time and thus maximize the chances of therapeutic success. This is especially true in the initial phase of sepsis when cardiac output and capillary permeability increase and protein binding is altered; this leads to increased clearance and a larger volume of distribution, inducing low serum and tissue beta-lactams concentrations [1, 30]. Underexposure increases the likelihood of therapeutic failure and the emergence of resistance. Improving antibiotic exposure is, therefore, a major challenge. Secondly, organ dysfunctions (and especially kidney failure) lead to high serum antibiotic concentrations. CRRT may limit underdosing when using full doses with a potential risk of neurotoxicity in the event of overdosing [4, 12, 15]. The mechanisms by which antibiotics are eliminated by CRRT appear to be poorly understood since the majority of physicians do not reportedly adjust the dosage as a function of the dialysis dose or effluent flow.

Our survey highlighted the broad implementation of extended and continuous infusions and so showed that physicians were well aware of the time-dependent nature of the beta-lactams’ activity. Even though the guidelines recommending the use of extended and continuous infusions for all compounds (to increase the probability of target attainment), these modalities are still mainly used for a few beta-lactams only and adherence to guidelines is suboptimal [5, 20]. This might be due to the recent changes in the French guidelines between 2014 and 2018. The French-speaking Intensive Care Society initially recommended continuous infusions for ceftazidime and extended infusions (over 3-4 h) for a few other beta-lactams [31]. In October 2018, the French Society of Anesthesia and Intensive Care Medicine and the French Society of Pharmacology and Therapeutics suggested the use of prolonged or continuous infusion of beta-lactams (intending to increase the probability of PK/PD target attainment and clinical cure rates) but did not differentiate between the various molecules [5]. These guidelines apply to infections with high-MIC bacteria or with non-fermenting Gram-negative bacilli, in patients in shock or with high severity scores, and lower respiratory tract infections (as described in our clinical vignettes). However, these guidelines were published just a few months before our survey, which may have limited their dissemination. Continuous and extended infusions are mostly used for piperacillin-tazobactam and ceftazidime; these are the compounds for which we have the most literature data, as reported in the ANTIBIOPERF study performed in 2015 [32]. Continuous infusion is mentioned in the French summary of product characteristics for ceftazidime only [33]. In the last decade, several other surveys have focused on these practices. A survey of 34 Belgian hospitals in 2011 showed that four beta-lactams were administered in the ICU by continuous and extended infusion to a varying extent: in 35% of the ICUs for cefepime, 38% for piperacillin-tazobactam, 68% for meropenem, and 81% for ceftazidime [34]. In 2013, an international multicenter survey of 402 physicians in 53 countries reported that piperacillin/tazobactam and carbapenem (meropenem and imipenem) were mainly administered as intermittent infusions (71% and 68%, respectively) [35]. In 2019, a German study reported that meropenem (70%), piperacillin/tazobactam (67%) and imipenem (50%) were the beta-lactams most regularly administered as prolonged and continuous infusions [36]. A survey performed in Australia and New Zealand in 2016 focused on meropenem and piperacillin-tazobactam, which were most frequently administered in intermittent infusions [37]. The administration method may therefore differ from one geographic region to another. The survey of Australia and New Zealand highlighted the fact that the infusion modality could be determined on a case-by-case basis, depending on the presence of bacteria with high MICs, pathological changes (sepsis or sepsis shock), and the severity of the patient’s illness [37].

The prescription tools used by the majority of physicians are not designed to recommend personalized dosages, and the use of pharmacokinetic software for finer dosage adjustment is rare. However, beta-lactam TDM is increasingly being used in CRRT, in line with the guidelines [5, 20]. The 2018 French guidelines suggested performing TDM for ICU patients with expected variability in beta-lactam pharmacokinetics and/or in patients with clinical signs potentially related to beta-lactam toxicity [5]. The 2020 European guidelines recommended TDM of beta-lactams as a standard of care in critically ill adult patients [20]. The fact that 56% of the physicians in our survey reported using full beta-lactam doses during CRRT and 62% mentioned using TDM suggests that beta-lactam TDM is often used only to determine the etiology of the neurotoxicity and that poor availability of TDM technique and the long wait for results from subcontractors limits the use of TDM in practice. This limitation has also been highlighted by other surveys [32, 35, 36, 38]. TDM requires complex analytical systems (such as high-performance liquid chromatography or liquid chromatography/mass spectrometry) associated with high equipment and personnel costs. TDM is mostly performed for meropenem (22%) and piperacillin (17%) but is available for other beta-lactams in less than 5% of hospitals [36]. A lack of access to TDM at the weekend, the perception of a wide therapeutic range, and the relatively recent awareness of the clinical implications of beta-lactam underdosing were major hindrances to implementation [35, 36]. Moreover, MICs for beta-lactams are not measured on a routine basis, which prevents antibiotic therapy from being adapted accordingly. Indeed, European Committee on Antimicrobial Susceptibility Testing (EUCAST) breakpoints are reported by laboratories in categories (i.e. susceptible, intermediate, or resistant) [36, 38]. An international survey of 328 hospitals in 53 countries performed in 2013 showed that TDM of piperacillin/tazobactam and meropenem was rare [35]. A French survey performed in 2015 found that beta-lactam TDM was available to 21% of the 507 respondents [32]. In 2019, a German survey showed that piperacillin, meropenem and ceftazidime were the beta-lactams most commonly dosed with TDM - especially in patients undergoing RRT [38]. Overall, these surveys highlighted differences in beta-lactam TDM practices, patient selection, PK/PD targets, drug assay methods, and dose adjustment strategies.

The diversity of PK/PD targets emphasizes the uncertainties in the literature data, even though most physicians are well aware that a beta-lactam concentration over several times the MIC is required throughout the dosing interval (as already reported by Wong et al. in 2014 [38]). However, few of our respondents answered this question, showing that the concept of dosage adjustment based on PK/PD indices is poorly known (as already described in the ONTAI study [36]). In 2018, the French Society of Pharmacology and Therapeutics and the French Society of Anesthesia and Intensive Care Medicine suggested that targeting a free plasma beta-lactam concentration over four times the MIC of the causative bacteria (or the EUCAST epidemiological cut-off, when the MIC of the isolated strain is not available) for 100% of the dosing interval would maximize the bacteriological and clinical responses in critical care patients, whereas the European guidelines recommend a PK/PD index of between two and five times the MIC [5].

The duration of the beta-lactam infusion was not defined in the questionnaire’s clinical vignettes; this limited the interpretation of our respondent’s practices but highlighted the diversity of dosing regimens used. Although meropenem is the best-studied beta-lactam in CRRT, its dosing regimens differed most significantly among our respondents (Table 1, Supplementary material). This variability was also evidenced in a Belgian study of ICUs and non-ICU wards [34].

Moreover, our survey results underlined the need for procedures and the importance of a multidisciplinary approach for providing stable infusions, since most physicians are not aware of the stability data [32, 34]. The fact that respondents using TDM were more likely to call other specialists and more likely to use prolonged/continuous infusions shows that TDM is part of a comprehensive, complex approach to PK/PD optimization, which also requires determination of the MIC and knowledge of the PK/PD target. The TDM included in Bayesian software represents the best option for personalizing antimicrobial dosing by taking account of various parameters (the MIC, site of infection, weight, renal function, severity, etc.). However, not all the clinical scenarios are available, and the high level of sophistication of these pharmacokinetic tools limits their implementation. Further studies are required to validate these tools and their potential clinical impact. Moreover, the variety of selected PK/PD targets raises the question of whether TDM is useful, given the resulting differences in dose adjustments. The different perceptions of neurotoxicity in patients on CRRT are probably related to the diversity of doses used, as illustrated in the replies to the clinical vignettes.

Our survey had some limitations. Firstly, the questionnaire did not fully reflect the complexity of having to decide on the dosing regimen at the bedside. The clinical vignettes and questions were simple and standardized. Secondly, the low response rate (11%) was explained by recruitment bias, since the survey was conducted during the summer vacation. Thirdly, we did not have an exhaustive list of intensivists in France. However, all types of hospitals (university or not, public or private sector, etc.) were represented. Despite these limitations, we undertook the largest yet study of this type in French ICUs. The diversity of replies to our questionnaire highlights the uncertainties regarding dosage adjustments required in CRRT and the lack of harmonization of PK/PD targets and emphasized the need for further research on a topic that is crucial in critically ill patients.

## Conclusions

The diversity of beta-lactam dosing regimens and the tools used to adjust it may be responsible for significant morbidity and mortality. Optimal antimicrobial dosing during CRRT remains challenging. Personalized TDM and the use of Bayesian softwares appear to be fundamental for optimizing beta-lactam dosing regimens and improving patients’ outcomes. However, low availability and a lack of clinical validation limit the implementation of these tools.

## Supplementary Information

**Additional file 1.**

## Data Availability

The datasets used and/or analysed during the current study are available from the corresponding author on reasonable request.

## Abbreviations

CRRT: Continuous renal replacement therapy
EUCAST: European Committee on Antimicrobial Susceptibility Testing
ICU: Intensive care unit
IQR: Interquartile range
KDIGO: Kidney Disease:Improving Global Outcomes
MIC: Minimal inhibitory concentration
PK/PD: Pharmacokinetic/pharmacodynamic
RRT: Renal replacement therapy
TDM: Therapeutic drug monitoring
