# Supplementary material for: Beta-lactam dosing during continuous renal replacement therapy: a survey of practices in french intensive care units
Source: BMC Nephrol. 2022 Jan 29;23:48. doi: 10.1186/s12882-022-02678-x (PMC8800323; doi:10.1186/s12882-022-02678-x)
Supplement: Supplementary file 1 — Additional file 1. [file 12882_2022_2678_MOESM1_ESM.doc]

# Supplementary material

**Supplement to :**

**Beta-lactam dosing during continuous renal replacement therapy: a survey of French physicians’ practices.**

Elodie Matusik, Justine Lemtiri, Guillaume Wabont, Fabien Lambiotte.

**Journal :** *BMC Nephrology*

**Corresponding author :**

Elodie Matusik, Department of Pharmacy and Department of Intensive Care Unit, Valenciennes General Hospital, Valenciennes, France. Tel : 0033-637942260. E-mail : [elodie.matusik@gmail.com](mailto:elodie.matusik@gmail.com), ORCID : 0000-0003-3046-145X.

Table 1: Meropenem pharmacokinetics data in continuous renal replacement therapy

**Table 1 :** **Meropenem pharmacokinetics data in continuous renal replacement therapy**

| **Author, Year** | **Pharmacokinetic model** | **Type of RRT, No. of patients (n=)** | **Filter material,  Surface area (m²)** | **Total effluent flow rate** | **Meropenem dose** | **Sieving or saturation coefficient** | **Volume of distribution** | **Total clearance (L/h)** | **CRRT clearance (L/h)** | **Target concentration (mg/L)** | **Dose recommended by author** |
| --- | --- | --- | --- | --- | --- | --- | --- | --- | --- | --- | --- |
| Krueger, 1998 [1] | Bicompartimental | CVVHDF (9) | AN69,  0.9 | 1.74 L/h | 1g/12h II | 1.06 | 0.26  ± 0.09 L/kg | 3.28 ± 1.02 | 3.19 | C 100% T > 4 and 8 | 1g/12h |
| Thalhammer, 1998 [2] | Monocompartimental | CVVH (9) | Polysulfone, 0.43 | 2.75 L/h | 1g II as a single dose | 1.09 ± 0.1 | 29.5 ± 2.7 L | 8.62 ± 1.12 | 2.98 ± 0.5 | C* 50% T > 12 | 1g/8h II |
| Tegeder, 1999 [3] | Monocompartimental | CVVH (9) | AN69,  / | 1.1-1.15 L/h | 0.5g/8-12h II | 1.17 ± 0.11 | 12.4 ± 1.8 L | 3.12 ± 0.5 | 1.32 ± 0.28 | Css 100% T > 12 | 0.5g/12h or 0.25g/6h II |
| Meyer, 1999 [4] | Monocompartimental | CVVHDF (1) | AN69,  / | 2 L/h | 1g/8-12h II | 1.02-1.13 | 0.54-0.63 L/kg | 7.76-8.47 | 1.2 | Css 100% T > 0.016 | 1g/12h II |
| Valtonen, 2000 [5] | Monocompartimental | CVVH or CVVHDF (6) | Polysulfone, 0.7 | CVVH :  0.4 L/h or CVVHDF : 1.4 or 2.4L/h | 1g II as a single dose | / | / | CVVH : 7.6 CVVHDF 1.4 L/h : 4.7  CVVHDF 2.4 L/h : 3.71 | / | C 100% T > 8 | CVVHDF : 1g/12h II CVVH : 0.5g/8h II |
| Giles, 2000 [6] | Bicompartimental | CVVH or CVVHDF (10) | AN69,  0.9 | CVVH :  1.7 L/h CVVHDF : 2.4 L/h | 1g/12h II | 0.93 | 27.3 L ± 9.8 = 0.35 L/kg ± 0.1 | CVVH : 3.88 CVVHDF : 4.72 | CVVH : 1.5 CVVHDF : 2.33 | Css 67% T > 4 | 1g/12h for susceptible bacteria Higher dosage regimens if MIC > 4 mg/L |
| Ververs, 2000 [7] | Monocompartimental | CVVH (5) | AN69,  / | 1.5-1.8 L/h | 0,5g/12h II | 0.63 ± 0.25 | 0.37 L/kg  ± 0.15 | 4.57 ± 0.89 | 1.03 ± 0.42 | C* 100% T > 1 | 0,5g/12h II for susceptible bacteria ↘ dose interval if intermediate susceptible strain |
| Krueger, 2003 [8] | Bicompartimental | CVVH (8) | AN69,  0.9 | 1.6 L/h | 0.5g/12h II | 0.91 ± 0.1 | 0.28 L/kg | 4.98 ± 1.3 | 1.46 ± 0.48 | Css 40% T > 4 | 0.5g/12h for susceptible bacteria 1g/12h if intermediate strains or if effluent flow > 1.6 L/h |
| Robatel, 2003 [9] | Quadricompartimental | CVVHDF (13) | AN69,  0.9 | 1.7 L/h | 0.5-1g/8-12h II | 0.65 | 33.2 L | 4.48 | 1.62 | C 75% T > 4 | 0.75g/8h or 1.5 g/12h II |
| Isla, 2005 [10] | Monocompartimental | CVVH or CVVHDF (20) | Polysulfone, 1.4  or AN69,  0.9 | Anuric patients : 1.5-2.5 L/h If GFR 10-50 mL/min : 2-2.8 L/h If GFR > 50 mL/min :  1-2.5 L/h | 0.5g/6-8h II 1g/6-8h II 2g/8h II | If anuria : 0.76 If GFR 10-50 mL/min : 0.85 If GFR > 50 mL/min : 0.72 | If anuria : 0.57 L/kg If GFR 10-50 mL/min : 0.37 L/kg If GFR > 50 mL/min : 0.44 L/kg | If anuria : 9 If GFR 10-50 mL/min : 8.1  If GFR > 50 mL/min : 63.89 | If anuria : 1.62 If GFR 10-50 mL/min : 1.93 If GFR > 50 mL/min : 0.98 | Css* 100% T > 2 à 4 | If anuria : 0.5g/8h to target 100% T > 4 mg/L  If GFR 10-50 mL/min 1g/8h to target 100% T > 2 mg/L Si GFR > 50 mL/min : dose regimens > 2g/8h |
| Langgartner, 2008 [11] | Monocompartimental | CVVHDF (6) | Polysulfone, 1.4 | 25 mL/kg/h | LD 0.5g and 1g/12h II LD 0.5g and 2g/24h CI | II : 0.97 (0.87-1.05) CI : 0.89 (0.79-0.93) | 32.3 L  (28.9-40.7) | II : 4.32 CI : 4.4 | / | Css 100% T > 8 | LD 0.5g then 2g/24h CI |
| Bilgrami, 2010 [12] | Monocompartimental | CVVH (10) | AN69,  2.15 | 4.4 L/h | 1g/8h II | 0,93 | 0.37 L/kg (0.32-0.46) | 6.0 (5.2 – 6.2) | 3.5 (3.4-3.9) | Css 100% T > 4 | 1g/8h II |
| Seyler, 2011 [13] | Monocompartimental | CVVH or CVVHDF (17) | AN69,  / | 45 mL/kg/h | 1g/12h II | / | 0.45 L/kg (0.20-3.03) | 4.83 | / | Css 100% T > 8 | 1g/8h II during 48h then dose reduction |
| Afshartous, 2014 [14] | Monocompartimental | CVVHD (10) | / | 23.4 ± 7.4 mL/kg/h | / | / | 26.9 L | 3 | 2.08  (1.45-2.41) | C* 100% T > 8 | LD then dose reduction |
| Beumier, 2014 [15] | Monocompartimental | CVVH or CVVHDF (25) | Polysulfone or AN69,  / | < 48h : 36.14 mL/kg/h > 48h : 34.2 mL/kg/h | 1g/8h II | / | 33.3 L  (9.2-86.5)  = 0.39 L/kg (0.11-1.20) | 4.32-5.9 (1.28-16.92) | / | Css 40% T > 8 | 1g/8h II |
| Varghese, 2015 [16] | Monocompartimental | CVVHDF | AN69,  1.05 | 2-3 L/h | 0.5g/8h II | Day 2 : 1.08 (0.95–1.15)  Day 5 : 1.01 (0.76–1.18) | V_day2_ : 0.35 (0.25–0.46)  V_day5_ : 0.33 (0.30–0.36) | Cl_day2_ : 4.1 (4.1–4.8)  Cl_day5_ : 3.8 (3.2–4.8) | Cl_day2_ : 2.9 (2.7–3.1)  Cl_day5_ : 2.8 (2.3–3.3) | Css % T > 2-16 | 0.5g/8h |
| Kawano, 2015 [17] | Bicompartimental | CVVHD (4) | Polysulfone, / | 0.7 L/h | 0.5g/8-12h II 1g/12h II | / | V1 : 17.5 ± 5.6 L | 2.95 | / | Css 40% T > 16 | 0.5g/8h or 1g/8-12h |
| Jamal, 2015 [18] | Monocompartimental | CVVH (16) | Polysulfone, 1.2 | 2 L/h = 30.09 mL/kg/h  (25–33.33 mL/kg/h) | LD 1g then 3g/24h CI LD 2g then 1g/8h II | II : 1.08-1.21 CI : 0.94-0.92 | 0.43 L/kg (0.40-0.50) | II : 0.98; IIss : 1.29 CI : 0.98; CIss :1.12 | II : 0.58 L/h ; CIss : 0.66 L/h CI : 0.49 L/h ; CIss : 0.49 L/h | II : C 40% T > 8 CI : C 100% T > 10 | Lower doses than LD 1g then 3g/24h CI or LD 2g then 1g/8h for susceptible bacteria with MIC < 2 mg/L but CI required in less susceptible bacteria |
| Ulldemolins, 2015 [19] | Monocompartimental  Population pharmacokinetics | CVVHDF (26) or CVVH (4) | AN69,  1.5  or AN69,  0.9 | 34.7 mL/kg/h  (18.7-60.1 mL/kg/h) | 0.5g/8-12h II 0.5g/6-8h EI 3h 1g/8-12h II ; 1g/8-12h  EI 3-4h ; 2g/8h II | / | 33 L | 3.68 ± 0.22 | / | Css* 40% T > 4 or  Css* 100% T > 4 or  Css* 5 x 100% T > 4 | If 40% T > 1 mg/L : 0.5g/8h II If 100% T > 1 mg/L : 0.5g/8h EI 3h  If 100% T > 4 mg/L : 500 mg/6h II or EI 3h if diuresis is preserved  If 100% T > 5 mg/L : 1g/8h EI 3h |
| Roberts, 2015  Roberts, 2012 [20-21] | Monocompartimental | CVVHDF (17) | AN69,  1.2 | 25 or 40 mL/kg/h | 0.5-1g/8-12h II | / | 17 L = 0.14-0.61 L/kg | 2.28  (1.38-5.70) | 1.38 L/h to 25 mL/kg/h and 1.68 L/h to 40 mL/kg/h | C* > 4 | / |
| Burger, 2018 [22] | Bicompartimental  Population pharmacokinetics | CVVHDF (49 including 15 patients of Robatel’s study) | Polysulfone, 1.2 | / | / | 0.75 | V1 = 17 L  V2 = 16 L | 4.8 (3.5–6.3) | / | Css 100% T > 2 or  Css 100% T > 8 | If Css 100% T > 2 mg/L :  1.5 g/12h II If Css 100% T > 8 mg/L : 1g/8h II insufficient,  prefer 2g/24h CI |
| Le Noble [23] 2019 | Monocompartimental | CVVH | AN69,  1.0 | 3.04 L/h | 1g LD then 2-3 g/24h CI | 0.45-0.96 | 19.9 L (18.8-20.8) | 1.49 mL/kg/min (0.84-2.17) | 0.45 mL/kg/min (0.31-0.55) | Css 100% T > 4 | 2-3g/24h CI |
| Padullés Zamora, 2019 [24] | Bicompartimental  Population pharmacokinetics | CVVHDF (12) | AN69,  1.5 | 3 L/h | 1g/8h II | 0.7 | V1 = 24,9 L V2 = 283 L | 7.78 | 6,49 | Css 100% T > 2 or  Css 100% T > 8 | If 100% T > 2 mg/L :  0.5g/8h II or CI  If 100% T > 8 mg/L : 3g/24h CI or 2g/8h EI 2h or CI |
| Onichimowski, 2020 [25] | Bicompartimental  Population pharmacokinetics | CVVH (9) or CVVHD (10) | Polysulfone, 1.8 | CVVHD : 8.16 L/h (6.6-9.6) CVVH : 35 mL/kg/h | 1g/8h EI 1h | / | V1=27.9 L  V2=33.7L | 21.1 | 15.1 | Css* 40% T > 2 or  Css 100% T > 2 | If 40% T > 2 mg/L : 1g/8h EI 1h but insufficient for 100% T > 2 mg/L |

Abbreviations : II : intermittent infusion, EI : extended infusion, CI : continuous infusion, LD : loading dose, C* : free concentration, C : total concentration, Css : steady state concentration, AN69 : polyacrylonitrile, GFR : glomerular filtration rate

**References**

1. Krueger WA, Schroeder TH, Hutchison M, et al. Pharmacokinetics of meropenem in critically ill patients with acute renal failure treated by continuous hemodiafiltration. Antimicrob Agents Chemother. 1998;42(9):2421-4. doi: 10.1128/AAC.42.9.2421.

2. Thalhammer F, Schenk P, Burgmann H, et al. Single-dose pharmacokinetics of meropenem during continuous venovenous hemofiltration. Antimicrob Agents Chemother. 1998;42(9):2417-20. doi: 10.1128/AAC.42.9.2417.

3. Tegeder I, Neumann F, Bremer F, et al. Pharmacokinetics of meropenem in critically ill patients with acute renal failure undergoing continuous venovenous hemofiltration. Clin Pharmacol Ther. 1999;65(1):50-7. doi: 10.1016/S0009-9236(99)70121-9.

4. Meyer MM, Munar MY, Kohlhepp SJ, et al. Meropenem pharmacokinetics in a patient with multiorgan failure from Meningococcemia undergoing continuous venovenous hemodiafiltration. Am J Kidney Dis. 1999;33(4):790-5. doi: 10.1016/s0272-6386(99)70236-2.

5. Valtonen M, Tiula E, Backman JT, et al. Elimination of meropenem during continuous veno-venous haemofiltration and haemodiafiltration in patients with acute renal failure. J Antimicrob Chemother. 2000;45(5):701-4. doi: 10.1093/jac/45.5.701.

6. Giles LJ, Jennings AC, Thomson AH, et al. Pharmacokinetics of meropenem in intensive care unit patients receiving continuous veno-venous hemofiltration or hemodiafiltration. Crit Care Med. 2000;28(3):632-7. doi: 10.1097/00003246-200003000-00005.

7. Ververs TF, Van Dijk A, Vinks SA, et al. Pharmacokinetics and dosing regimen of meropenem in critically ill patients receiving continuous venovenous hemofiltration. Crit Care Med. 2000;28(10):3412-6. doi: 10.1097/00003246-200010000-00006.

8. Krueger WA, Neeser G, Schuster H, et al. Correlation of meropenem plasma levels with pharmacodynamic requirements in critically ill patients receiving continuous veno-venous hemofiltration. Chemotherapy. 2003;49(6):280-6. doi: 10.1159/000074527.

9. Robatel C, Decosterd LA, Biollaz J, et al. Pharmacokinetics and dosage adaptation of meropenem during continuous venovenous hemodiafiltration in critically ill patients. J Clin Pharmacol. 2003;43(12):1329-40. doi: 10.1177/0091270003260286.

10. Isla A, Maynar J, Sánchez-Izquierdo JA, et al. Meropenem and continuous renal replacement therapy: in vitro permeability of 2 continuous renal replacement therapy membranes and influence of patient renal function on the pharmacokinetics in critically ill patients. J Clin Pharmacol. 2005;45(11):1294-304. doi: 10.1177/0091270005280583.

11. Langgartner J, Vasold A, Glück et al. Pharmacokinetics of meropenem during intermittent and continuous intravenous application in patients treated by continuous renal replacement therapy. Intensive Care Med. 2008;34(6):1091-6. doi: 10.1007/s00134-008-1034-7.

12. Bilgrami I, Roberts JA, Wallis SC, et al. Meropenem dosing in critically ill patients with sepsis receiving high-volume continuous venovenous hemofiltration. Antimicrob Agents Chemother. 2010;54(7):2974-8. doi: 10.1128/AAC.01582-09.

13. Seyler L, Cotton F, Taccone FS, et al. Recommended β-lactam regimens are inadequate in septic patients treated with continuous renal replacement therapy. Crit Care. 2011;15(3):R137. doi: 10.1186/cc10257.

14. Afshartous D, Bauer SR, Connor MJ, et al. Pharmacokinetics and pharmacodynamics of imipenem and meropenem in critically ill patients treated with continuous venovenous hemodialysis. Am J Kidney Dis. 2014;63(1):170-1. doi: 10.1053/j.ajkd.2013.08.015.

15. Beumier M, Casu GS, Hites M, et al. β-lactam antibiotic concentrations during continuous renal replacement therapy. Crit Care. 2014;18(3):R105. doi: 10.1186/cc13886.

16. Varghese JM, Jarrett P, Wallis SC, et al. Are interstitial fluid concentrations of meropenem equivalent to plasma concentrations in critically ill patients receiving continuous renal replacement therapy? J Antimicrob Chemother. 2015;70(2):528-33. doi: 10.1093/jac/dku413.

17. Kawano S, Matsumoto K, Hara R, et al. Pharmacokinetics and dosing estimation of meropenem in Japanese patients receiving continuous venovenous hemodialysis. J Infect Chemother. 2015;21(6):476-8. doi: 10.1016/j.jiac.2015.02.011.

18. Jamal JA, Mat-Nor MB, Mohamad-Nor FS, et al. Pharmacokinetics of meropenem in critically ill patients receiving continuous venovenous haemofiltration: a randomised controlled trial of continuous infusion versus intermittent bolus administration. Int J Antimicrob Agents. 2015;45(1):41-5. doi: 10.1016/j.ijantimicag.2014.09.009.

19. Ulldemolins M, Soy D, Llaurado-Serra M, et al. Meropenem population pharmacokinetics in critically ill patients with septic shock and continuous renal replacement therapy: influence of residual diuresis on dose requirements. Antimicrob Agents Chemother. 2015;59(9):5520-8. doi: 10.1128/AAC.00712-15.

20. Roberts DM, Roberts JA, Roberts MS, et al. RENAL Replacement Therapy Study Investigators. Variability of antibiotic concentrations in critically ill patients receiving continuous renal replacement therapy: a multicentre pharmacokinetic study. Crit Care Med. 2012;40(5):1523-8. doi: 10.1097/CCM.0b013e318241e553.

21. Roberts DM, Liu X, Roberts JA, et al. RENAL Replacement Therapy Study Investigators. A multicenter study on the effect of continuous hemodiafiltration intensity on antibiotic pharmacokinetics. Crit Care. 2015;19(1):84. doi: 10.1186/s13054-015-0818-8.

22. Le Noble JLML, Meenks SD, Foudraine N, et al. Alterations in transmembrane pressures during continuous venovenous haemofiltration significantly contribute to the pharmacokinetic variability of meropenem: a case series of three patients. J Antimicrob Chemother. 2019;74(1):271-273. doi: 10.1093/jac/dky411.

23. Burger R, Guidi M, Calpini V, et al. Effect of renal clearance and continuous renal replacement therapy on appropriateness of recommended meropenem dosing regimens in critically ill patients with susceptible life-threatening infections. J Antimicrob Chemother. 2018;73(12):3413-3422. doi: 10.1093/jac/dky370.

24. Padullés Zamora A, Juvany Roig R, Leiva Badosa E, et al. Optimized meropenem dosage regimens using a pharmacokinetic/pharmacodynamic population approach in patients undergoing continuous venovenous haemodiafiltration with high-adsorbent membrane. J Antimicrob Chemother. 2019;74(10):2979-2983. doi: 10.1093/jac/dkz299.

25. Onichimowski D, Będźkowska A, Ziółkowski H, et al. Population pharmacokinetics of standard-dose meropenem in critically ill patients on continuous renal replacement therapy: a prospective observational trial. Pharmacol Rep. 2020;72(3):719-729. doi: 10.1007/s43440-020-00104-3.
